# Tricuspid Valve Replacement, Mechnical vs. Biological Valve, Which Is Better?

**Published:** 2013-06-01

**Authors:** Haitham Akram Altaani, Saed Jaber

**Affiliations:** 1 Department of Cardiac Surgery, Queen Alia Heart Institute, Royal Medical Services, Amman, Jordan

**Keywords:** Tricuspid Regurgitation, Tricuspid Valve, Valve Repair

## Abstract

**Background:**

The initial trial in tricuspid surgery is repair; however, replacement is done whenever the valve is badly diseased. Tricuspid valve replacement comprises 1.7% of all tricuspid valve surgeries.

**Materials and Methods:**

The present retrospective study was performed using the medical records of 21 cases who underwent tricuspid valve replacement from January 2002 until the end of December 2010. The mean age of the participants was 52.3±8.8 years and 66.7% were females. In addition, tricuspid valve replacement was associated with mitral valve surgery, aortic valve surgery, and both in 14.3%, 4.8%, and 33.3% of the cases, respectively. Yet, isolated tricuspid valve replacement and redo surgery were performed in 10 cases (47.6%) and 8 cases (38.1%), respectively. Besides, trial of repair was done in 14 cases (66.7%). Moreover, biological and mechanical valves were used in 76.2% and 23.8% of the patients, respectively.

**Results:**

According to the results, early mortality was 23.8% and one year survival was 66.7%. Moreover, early mortality was caused by right ventricular failure, multiorgan failure, medistinitis, and intracerbral bleeding in 42%, 28.6%, 14.3%, and 14.3% of the cases, respectively. In addition, 57.1% of the deaths had occurred in the cases where the biological valve was used, while 42.9% of the deaths had taken place where the mechanical one was utilized.

**Conclusions:**

The patients who require tricuspid valve replacement are usually high risk surgical candidates with early and long term mortality. The findings of the current study showed no significant hemodynamic difference between mechanical and biological valves.

## 1. Introduction

Tricuspid valve replacement is the second and the last choice in tricuspid valve surgery. The choice to insert mechanical or bioprosthetic valve remains controversial. In the present study, we analyzed the cases which underwent tricuspid valve replacement at Queen Alia heart institute from 2002 to 2010. Then, we discussed the results of both mechanical and bioprosthatic replacements compared to other studies conducted on the issue.

## 2. Materials and Methods

From January 2002 until the end of December 2010, 633 tricuspid valve surgeries were performed at Queen Alia heart institute. Among these cases, 21 ones (3.32%) were replaced, while the others were repaired by different types of tricuspid valve repair. Among the replaced valve patients, 14 ones (66.7%) were female and their mean age was 52.3+8.8 years. In addition, 10 cases (47.6%) underwent isolated tricuspid valve replacement, while the other 11 cases (52.4 %) underwent other cardiac procedures; mitral and aortic valve, mitral valve, and aortic valve surgery in seven (33.3%), three (14.2%), and one patient (4.8 %), respectively. Furthermore, eight patient (38.1%) underwent the surgery as a second procedure (redo surgery); 3, 3, and 2 patients having the history of mitral surgery alone, mitral with tricuspid repair, and isolated tricuspid valve replacement, respectively . The etiology of the tricuspid valve was sever regurgitation of tricuspid valve with unhealthy leaflets in 10 cases (47.6%), tricuspid valve endocarditis in 6 cases (28.6%), previously replaced valve in 2 cases (9.5%), gross regurgitation over the previously repaired valve in one case (4.8%), and severely stenosed valve over the previously repaired valve in 2 cases (9.5%). The initial trial of the repair was done in 14 cases (66.7%) before making decision for replacement. Besides, biological prosthetic valve was inserted in 16 patients (76.2%), while the mechanical valve was inserted in the other 5 patients (23.8 %). It should be mentioned that the decision to insert the biological prosthetic or mechanical valve was based on the patients’ age (mechanical and biological valves in the patients below and above 65 years old, respectively) and the type of other inserted valves. In addition, biological prostheses were utilized in isolated tricuspid valve replacement regardless of age. Biological prostheses were also used in case of any contraindications for anticoagulants (Table 1). 

**Table 1.** Preoperative and Intraoperative Data

Variable	mean±SD / n(%)
Age	52.20±8.8
Sex	
Female	14(66.7%)
Male	7(33.3%)
Associated procedures	
Mv[Table-fn fn4993]	3(14.3%)
Av[Table-fn fn4993]	1(4.8%)
Both	7(33.3%)
Isolated	10(47.6%)
Redo surgery	8 (38.1%)
Intraoperative findings	
Stenosed valve	2 (9.5%)
Regurgitant repaired	1 (4.8%)
Previously replaced	2 (9.5%)
Gross secondary regurge	10(47.6%)
Endocarditis	6(28.6%)
Trial of repair	14 (66.7%)
Valve used	
Biological	16 (76.2%)
Mechanical	(23.8%)

Abbreviations: MV, mitral valve; AV, aortic valve; TVR, tricuspid valve replacement

A midsternotomy incision was used in all the patients. Moreover, cardiopulmonary bypass was instituted through direct aortic cannulation in 19 cases, femoral artery cannulation in 2 cases and double venous cannulation followed by institution of total cardiopulmonary bypass by snaring of inferior and superior vena cava in all the cases. Also, replacement was done during the cardiac arrest in 4 cases and was achieved by antegrade crystalloid and/or blood cardioplegia at 28­32 ºC in addition to topical cooling with ice slashed normal saline 0.9%. In the other 17 cases, however, the replacement was done in the beating heart with total cardiopulmonary bypass. In case there were other valve procedures in any of the cases, the tricuspid valve replacement was performed after finishing the other valve.

Exploration of the valve was done through right atriotomy and cose groove retractor was used in all the cases. The leaflets of the tricuspid valve did not excise in 17 cases. They were excised in the cases with previously replaced valves or sever stenosis and fusion of all the leaflets. The prostheses were fixed by palgeted 2/0 ethebond horizontal mattress sutures in all the cases. In this study, 33, 31, and 28 mm prostheses were used in 11, 8, and 2 cases, respectively. After fixing the valve, the right atrium was closed by 5/0 polyproline continuous suture in 2 layers and the patients were weaned off the cardiopulmonary bypass. All the patients were kept on warfarine for 3 months in case of bioprosthetic valve and for whole life in case of mechanical valve in order to keep the International Normalized Ratio (INR) between 3 and 4.

## 3. Results

Early mortality occurred in 5 cases (23.8%). Among these cases, 2 could not be weaned off the cardiopulmonary bypass due to right ventricular failure and uncontrolled bleeding. It should be mentioned that these cases had previously underwent cardiac operation. Regarding the other three deaths, one case died after being transferred to ICU due to (RV) failure and malignant Arrhythmias, while the other 2 cases were reoperated for endocarditis and replaced with another valve, but died 5 days after the initial operation because of sever sepsis and Multiorgan failure (Table 2). 

**Table 2.** Postoperative Data

Variable	mean±SD / n(%)
Early mortality	23.8%
Late mortality	9.5%
One year survival	66.7%
Causes of death	
RV[Table-fn fn4994]failure	42.9%
MOF[Table-fn fn4994]	28.6%
Mediastinitis	14.3%
CVA[Table-fn fn4994]	14.3%
Redo surgery	8 (38.1%)
Intraoperative findings	
Stenosed valve	2 (9.5%)
Regurgitant repaired	1 (4.8%)
Previously replaced	2 (9.5%)
Gross secondary regurge	10(47.6%)
Endocarditis	6(28.6%)
Other complications	
Heart block	4.21(19%)
Wound infection	3.21(14.3%)
High INR	4.21(19%)
Mortality according to the valve	
Mechanical	3.7 (42%)
Biological	4.7(57.1%)
Functional class	
I-II	7.21(33.3%)
III-IV	7.21(33.3%)

Abbreviations: RV, right Ventricle; MOF, multiorgan failure; CVA, cerebrovascular accident

Overall, 3 of the patients who had undergone a previous operation died. Two cases receiving mechanical valve and 3 cases receiving bioprosthetic valve also died in this study. Moreover, the mortality rate was 4/10 (40%) in cases of isolated tricuspid valve surgery which might be due to the fact that most of them were in advanced situations with functional class III to IV ascitis, liver dysfunction, and previous cardiac operation (Table 2). 

Furthermore, 3 patients (14.2%) developed complete heart block. Thus, permanent pacemaker was intraoperatively inserted for these patients and they were all discharged from the hospital.

In this study, 11 patients were extubated in the first 24 hours after the operation and 6 patients were extubated after 24 hours. However, 5 patients were not extubated and died. The study results revealed one year survival as 14/21 (66.7%). The 2 last deaths were due to mediastinitis in one case and high INR in the other one who developed intracerbral bleeding.

The average follow-up period was 2 years. During this period, 1 and 3 cases were readmitted due to high INR and deep sternal wound infection, respectively. Nonetheless, no structural valve changes or high pressure gradient were detected in the patients during the follow up period. Furthermore, the functional class was II in 7, III in 4, and IV in 3 patients.

## 4. Discussion

 The initial choice for tricuspid surgery at our institute as well as other places is the repair; however, sometimes the valve has to be replaced because of sever structural valve dysfunction making it not amenable for repair. According to McGrath and colleagues, tricuspid valve operation comprises 5.7% of all the valvular interventions, with tricuspid valve replacement comprising 1.7% of all tricuspid valve surgeries (1). The patients who have undergone mitral and/or aortic valve surgery as a concomitant procedure in addition to tricuspid valve carry a high risk surgery and poor prognosis. In addition, early and late mortality rates in these patients have been reported as 27% and 12%, respectively (2).

Tricuspid valve replacement was associated with mitral valve surgery, aortic valve surgery, and both in 14.3%, 4.8%, and 33.3% of the cases, respectively. On the other hand, isolated tricuspid valve replacement was only performed in 47.6% of the cases. Besides, the early mortality rate was 23.8% and this high mortality rate might be because of the fact that the study patients had already been categorized as high risk surgical candidates, higher age group, concomitant surgeries, long duration of aortic cross clamp and cardiopulmonary bypass, presence of hepatic dysfunction, high mean pulmonary artery pressure, and right ventricular dysfunction (1,2).

The cause of death in most cases was the right ventricular dysfunction. Concomitant procedure (mitral, aortic, or both), having a history of previous cardiac surgeries (47%), multi organ failure, and intraoperative hemorrhage all could be the causes of death, as well (3).

Overall, the risk factors for death include preoperative edema, long duration of cross clam and cardiopulmonary bypass, high pulmonary artery pressure, previous cardiac surgery, being above 55 years old, and advanced preoperative functional class. Yet, right ventricular failure was the predominant cause of death in the current study (4).

The choice of valve for tricuspid replacement is controversial (1-3). Initially, mechanical prosthesis was used, but now it is replace by bioprosthetic valves in most of the cases (5). In fact, low pressure and stress in the right heart provides higher durability for the bioprosthesis compared to the left sided valves (1,6).

The advantage of the bioprosthetic valves is that they do not require long life anticoagulation in contrast to the mechanical valves and cause a higher risk for thromboembolism and hemorrhage (5-7). Mechanical valves, on the other hand, have desirable hemodynamic properties, low gradients, low disturbances in flow, and long durability.

The average time for tricuspid bioprosthesis failure is 7 years (4). We prefer bioprostheses because they do not require anticoagulation and, at the same time, have longer durability as well as lower reoperation rate compared to other left sided heart valve positions. They are also preferred whenever there is contraindication for anticoagulation, such as pregnancy and older age groups where the life expectancy is low (7-10). On the other hand, mechanical valves are preferred in younger age groups as well as the patients with another mechanical valve at the same time (7,9,10). Overall, the freedom from thromboembolism complications was 92.6% after 1 year.

Most of the studies have shown no significance differences between bioprosthetic and mechanical valves regarding hemodynamic parameters and early and late complications, which is similar to the results of the present study (1,9).

## 5. Conclusions

The patients who require tricuspid valve replacement are usually high risk surgical candidates with high early and late mortality. The most common cause of death was right ventricular failure in this study. However, the choice between mechanical and biological valves is still controversial.
